# Staff experiences of providing maternity services in rural southern Tanzania – a focus on equipment, drug and supply issues

**DOI:** 10.1186/1472-6963-13-61

**Published:** 2013-02-14

**Authors:** Suzanne Penfold, Donat Shamba, Claudia Hanson, Jennie Jaribu, Fatuma Manzi, Tanya Marchant, Marcel Tanner, Kate Ramsey, David Schellenberg, Joanna Armstrong Schellenberg

**Affiliations:** 1 Faculty of Infectious and Tropical Diseases, London School of Hygiene and Tropical Medicine, London, UK; 2 Ifakara Health Institute, Dar es Salaam, Tanzania; 3 Division of Global Health, Karolinska Institutet, Stockholm, Sweden; 4 Swiss Tropical and Public Health Institute, Basel, Switzerland and University of Basel, Basel, Switzerland

**Keywords:** Maternal health services, Neonate, Qualitative research, Equipment and supplies, Healthcare systems, Health manpower, Africa south of the Sahara

## Abstract

**Background:**

The poor maintenance of equipment and inadequate supplies of drugs and other items contribute to the low quality of maternity services often found in rural settings in low- and middle-income countries, and raise the risk of adverse patient outcomes through delaying care provision. We aim to describe staff experiences of providing maternal and neonatal care in rural health facilities in Southern Tanzania, focusing on issues related to equipment, drugs and supplies.

**Methods:**

Focus group discussions and in-depth interviews were conducted with different staff cadres from all facility levels in order to explore experiences and views of providing maternity care in the context of poorly maintained equipment, and insufficient drugs and other supplies. A facility survey quantified the availability of relevant items.

**Results:**

The facility survey, which found many missing or broken items and frequent stock outs, corroborated staff reports of providing care in the context of missing or broken care items. Staff reported increased workloads, reduced morale, difficulties in providing optimal maternity care, and carrying out procedures with potential health risks to themselves as a result.

**Conclusions:**

Inadequately stocked and equipped facilities compromise the health system’s ability to reduce maternal and neonatal mortality and morbidity by affecting staff personally and professionally, which hinders the provision of timely and appropriate interventions. Improving stock control and maintaining equipment could benefit mothers and babies, not only through removing restrictions to the availability of care, but also through improving staff working conditions.

## Background

Increasing utilisation of good quality maternity services is necessary to reduce maternal and newborn deaths and disability in low- and middle-income countries (LMIC) through the provision of skilled care, medication and clinical procedures, including emergency obstetric care (EmOC), to prevent and treat complications [1-5].

Health systems can only provide good quality maternity care if facilities have sufficient and skilled staff who have access to functioning equipment, and sufficient drugs and supplies [6]. The quality of maternity services is variable and often poor in LMIC [7-9]. Reasons for this include management issues [10], insufficient and inadequately skilled staff [10-12], high staff turnover and absenteeism [7,13] and poorly maintained equipment and stocks of drugs and other items [12,14]. The latter is itself an important direct contributor to the delay in women receiving timely and appropriate maternity care upon reaching a facility (known as the Phase 3 delay), which increases the risk of maternal and newborn mortality through delayed treatment of obstetric complications [15].

### Aim

Although previous studies have reported women’s views of their maternity care [16,17] and staff views regarding general working conditions [18], few have reported staff views of providing maternity care in LMIC, despite these being key to understanding staff motivation and working conditions.

Here we examine the experiences of professional staff providing maternal care in public rural health facilities in Southern Tanzania, focusing on issues arising in the context of poorly maintained equipment and insufficient key drugs and other supplies. We also aim to quantify the availability of functioning equipment and medical supplies.

### Tanzanian health system

The Tanzanian public health system comprises a network of dispensaries, health centres and hospitals. Health sector reform, since 1994, is characterized by decentralization with devolution [19], integration of vertical programs, promotion of private-public mix, and a sector wide approach. It also includes joint donor financing directly to districts through “basket funding” since 2001.

Council Health Management Teams (CHMTs) are responsible for health services in their district [20]. Each CHMT is headed by a District Medical Officer (DMO), who answers to the local government and is supported by the regional medical officer [19].

### Ordering and equipment maintenance systems

The purchase and distribution of drugs and equipment are mandated to the Medical Stores Department (MSD), a semiautonomous organisation under the Ministry of Health and Social Welfare [21]. Drug distribution changed in the mid 2000s to a “pull” system where facilities order according to their needs via the DMO’s office, which distributes items to facilities [21]. While facilities are allowed to purchase drugs from user fees and community health funds, lengthy bureaucracy undermines this option.

### Study context

The Tanzania demographic and health survey 2010 reported a maternal mortality ratio (MMR) of 454/100,000 deliveries and a neonatal mortality rate (NMR) of 26/1000 live births [22]; such levels warrant intensive reduction strategies. Southern Tanzania is largely rural: 39% of the population lives under the poverty line [23] and the MMR and NMR are higher than the national average (MMR: 731 in 2007 (unpublished data), NMR: 47 in 2004 [14]).

A national medicine supply assessment reported high availability of drugs in general (median availability of twenty tracer drugs in health facilities was 89%) but stock management was poor, with frequent and long-lasting stock outs [24]. For maternal care, a national health service provision assessment in 2006 showed there was much room for improvement [19]. Although antenatal care (ANC) was available in nearly all government facilities, fewer than half had all essential equipment, drugs and supplies for basic ANC, including blood pressure machine, foetoscope, and iron and folic acid tablets. While delivery services were widely available, basic items for conducting normal deliveries were available in only one in eight of those facilities.

The availability of items required for maternity care has been found to be lower in the Southern Zone of Tanzania compared to others nationally [19]. Despite this, assessments of the quality of reproductive health services in Lindi region in 1999 and 2007 [25] found improvements in the availability of equipment for maternal and neonatal care, such as scissors (availability increased from 34% of facilities in 1999 to 84% in 2007) and needle holders (increased from 23% in 1999 to 73% in 2007). Furthermore availability of some supplies greatly improved between the two surveys, e.g. syphilis test kits (from 0% to 54%) and intravenous kits (from 5% to 38%). Nevertheless, availability of other key items remained problematic, e.g. functioning blood pressure machines and working sterilisers were found in only half of facilities. Shortages and stock outs also remained common [25].

The availability of EmOC was well below international recommendations, often due to unavailability of parenteral anticonvulsants or assisted vaginal delivery [26]. Eighteen percent of hospitals provided comprehensive EmOC [19]. The coverage of EmOC in the Southern Zone of Tanzania was 0.7 facilities/500,000 people for basic emergency care, which is well below the United Nations recommended level of 4 facilities/500,000 people [19]. Data from 2000–2002 gave a rate for major obstetric interventions, essentially caesarean section, of 1.8% of expected births, indicating deficiencies in access to life-saving comprehensive EmOC in rural areas [27].

Despite these challenges, staff in rural health facilities in Tanzania provide ANC to the majority of pregnant women (95%) and assist many deliveries (41%) [22]. In the study area nearly all (99%) pregnant women attend ANC at least once [22], and over half of women deliver in a health facility [22,28], mainly public facilities [22].

## Methods

### Study area

The Improving Newborn Survival in rural Southern Tanzania (INSIST) project is developing, implementing and evaluating the effectiveness and cost of scaleable strategy interventions to improve neonatal survival. The study was conducted as part of INSIST in six districts of Lindi and Mtwara regions, which had a total population of about 1,000,000 people in 2007. The study setting is described elsewhere [13,29]. Briefly, the area has a wide mix of ethnic groups, including the Makonde, Mwera, and Yao. Common occupations include subsistence farming, fishing and small-scale trading. Most rural roads are unpaved, making some impassable in wet weather. HIV prevalence rates for adults age 15–49 years in Lindi and Mtwara regions were 3.8% and 3.6% respectively in 2007/8 [30].

### Study design

We conducted a mixed method study comprising a cross-sectional survey of all health facilities in the study districts, and qualitative focus group discussions (FGDs) and in-depth interviews (IDIs) with health managers and workers in order to understand the structure and function of health services in relation to maternal and newborn care.

### Data collection

#### Quantitative

A questionnaire, in Swahili, was developed with contributions from other publicly available tools such as the Safe Motherhood Needs Assessment [31], and with the addition of questions specific to the project. Questions allowed for a combination of staff-reported and interviewer-observed information, excluding exit interviews or case management observation. Data were collected on aspects of care provision including availability of drugs and supplies (restricted to presence or absence and recent stock outs), and availability and functioning state of equipment.

Data were collected in March 2009 by trained interviewers. Pairs of interviewers visited each of the 200 facilities in the six study districts without prior notice. If facilities were closed, revisits were not undertaken. Completed forms were reviewed in the evening after data collection and feedback given to interviewers. A subset of facilities was revisited to repeat a number of questions.

#### Qualitative

FGD [32] and IDI guides were designed to collect staff views on aspects of maternity care, focussing on maintenance of facility equipment and supplies of drugs and other items.

Between March and May 2009 seven FGDs were conducted with facility staff from government facilities (five with health centres and/or dispensary staff and two with hospital staff), and three IDIs were conducted with CHMT members. Facilities were purposively selected from three districts to reflect the geographical diversity of the study area. Health workers were selected purposively; those who provided maternal, newborn and child health services were invited to participate. Where more staff than required met the inclusion criterion facilities self-selected staff members to attend. From the three CHMTs that supervised the selected facilities, the reproductive and child health coordinator was invited to participate in an IDI. The number of FGDs conducted was determined using saturation sampling [32]; discussions were conducted until no new information was found.

Data were collected in Swahili by two male researchers who were trained in qualitative data collection methods, native Swahili speakers and fluent in English. All IDIs and FGDs were recorded, and notes written. Field notes were written up to expanded notes, which were the main data analysed. Recordings were accessed for clarification purposes only [33].

Data collection was entrenched the grounded theory [34-36]. All the transcripts were reviewed daily and the data used to inform the next FGD or IDI.

### Data processing and analysis

#### Quantitative

Pendragon Forms 4.0 software (http://pendragonsoftware.com/) was used to develop a modular questionnaire data entry template. For data collection, the questionnaire was loaded onto Palm m130 Personal Digital Assistants with 8 Mb RAM. Logical checks and skip patterns took place at data entry. Data records could not be edited after leaving each facility. Data were downloaded to laptop computers and daily summary reports produced to evaluate completeness. Data were analysed using Stata v10 (http://www.stata.com). We only included public health facilities as private and Mission facilities (n = 18) face different problems in relation to their specific procurement and staff support systems. We excluded facilities that did not provide antenatal or delivery services (n = 5). The equipment and drugs were checked against the required items for each facility level [37].

#### Qualitative

Expanded notes were explored through multiple readings to ensure familiarity with the data. Data were coded inductively using thematic content analysis by two independent researchers and consensus reached on the themes. The data were coded using NVivo v7 software (http://www.qrsinternational.com) and interpreted.

### Approvals and permission

The study was part of INSIST (http://www.clinicaltrials.gov, NCT01022788). The study was approved by the review boards of Ifakara Health Institute, the National Tanzanian Medical Research Co-coordinating Committee, Tanzania’s Commission for Science and Technology, and the London School of Hygiene and Tropical Medicine.

Prior written consent to approach the facilities to participate in the survey was obtained from each CHMT and a copy of the letter given to each facility before data collection started. Informed consent to participate, which assured anonymity and confidentiality, was obtained from FGD (oral) and IDI (written) participants.

## Results

### Facility characteristics

All 200 facilities in the study area were visited. Data could not be obtained from two owing to unavailability of staff due to poor weather and in-service trainings. Findings from the 175 government facilities (4 hospitals, 15 health centres and 156 dispensaries) providing antenatal or delivery services are shown here.

The majority of facilities had basic equipment for providing patient care, such as hand washing facilities (85%, Table 1). Over half (58%) of facilities had a fridge kept at 2–8°C on the day of survey. The availability of clinical equipment was generally lower: 61% of facilities had a blood pressure machine, 70% had an infant weighing scale and 57% had a speculum (Table 1). The availability of every item of equipment was highest in hospitals and lowest in dispensaries.

**Table 1:** Availability of equipment, drugs and supplies

**Item**	**n (%) facilities where item present (and functioning, for equipment)**				**N (%) facilities where broken equipment present**
	**Hospitals (N = 4)**	**Health centres (N = 15)**	**Dispensaries (N = 156)**	**Total (N = 175)**	**Total (N = 175)**
**Basic equipment**					
Examination couch	4 (100)	14 (93)	143 (92)	161 (92)	2 (1)
Storage area for drugs	4 (100)	15 (100)	142 (91)	161 (92)	3 (2)
Refrigerator (2–8 °C)	2 (50)	10 (63)	90 (58)	102 (28)	49 (28)
Hand washing facility	4 (100)	15 (100)	130 (83)	149 (85)	4 (2)
Telephone or transmitter	3 (75)	5 (33)	12 (8)	20 (11)	16 (9)
Electricity	4 (100)	15 (100)	53 (34)	72 (41)	5 (3)
Blood pressure machine	3 (75)	15 (100)	89 (57)	107 (61)	36 (21)
Adult weighing scale	4 (100)	15 (100)	118 (76)	137 (78)	18 (10)
Stethoscope	4 (100)	15 (100)	131 (84)	150 (86)	11 (6)
Means of sterilisation	3 (75)	15 (100)	139 (89)	157 (90)	5 (3)
Clinical thermometer	4 (100)	15 (100)	139 (89)	158 (90)	2 (1)
Scissors	4 (100)	14 (93)	142 (91)	160 (91)	5 (3)
**Equipment for maternal care**					
Fetoscope	4 (100)	15 (100)	149 (96)	168 (96)	2 (1)
Infant weighing scale	4 (100)	15 (100)	103 (66)	122 (70)	22 (13)
Transport for obstetric referral	4 (100)	8 (53)	30 (19)	42 (24)	7 (4)
Manual vacuum aspirator	4 (100)	7 (47)	1 (1)	12 (7)**	1 (1)
Speculum	4 (100)	15 (100)	81 (52)	100 (57)	7 (4)
Vacuum extractor	2 (50)	2 (13)	1 (1)	5 (3)	0 (0)
Needle holder	4 (100)	12 (80)	96 (62)	112 (64)	1 (1)
Adult ambu bag	2 (50)	2 (13)	3 (2)	7 (4)**	3 (2)
Newborn ambu bag	4 (100)	9 (60)	38 (24)	51 (29)	2 (1)
Suction machine + adult tubes	0 (0)	1 (7)	0 (0)	1 (1)	7 (4)
Suction machine + children tubes	2 (50)	1 (7)	5 (3)	8 (5)	0 (0)
**Supplies and drugs**					
Examination gloves	4 (100)	14 (93)	145 (93)	163 (93)	
Disposable syringes and needles – adult	4 (100)	10 (63)	125 (80)	139 (79)	
Giving set – blood	4 (100)	1 (7)	8 (5)	13 (7)	
Giving set – other fluids	4 (100)	15 (100)	92 (59)	111 (63)	
Intravenous cannulae – size 16, 18 or 20	4 (100)	12 (80)	57 (37)	73 (42)	
Protective clothing for staff	4 (100)	14 (93)	96 (62)	115 (66)	
Complete suture kits	3 (75)	8 (53)	44 (28)	55 (31)	
Normal saline	3 (75)	9 (60)	51 (33)	63 (36)	
Blank partograph	4 (100)	11 (73)	106 (68)	121 (69)	
Cord ligatures	4 (100)	11 (73)	122 (78)	137 (78)	
Disposable syringes and needles – neonate	4 (100)	10 (63)	130 (83)	144 (82)	
Oxygen cylinder	4 (100)	4 (27)	20 (13)	28 (16)	
Diazepam injection	4 (100)	14 (93)	126 (81)	144 (82)	
Magnesium Sulphate injection	3 (75)	8 (53)	80 (51)	91 (52)	
Hydralazine injection	3 (75)	0 (0)	2 (1)**	5 (3)	
Misoprostol tablets (100 or 200 μg)*	1 (25)	5 (33)	7 (4)	13 (7)	
Ergometrine injection	1 (25)	4 (27)	58 (37)	63 (55)	
Oxytocin injection	4 (100)	5 (33) **	23 (15)**	32 (18)	
Any antibiotic injection	4 (100)	14 (93)	127 (81)	145 (83)	
Ringer’s lactate	4 (100)	9 (60)	55 (35)	68 (39)	
10% Dextrose	3 (75)	1 (7)	2 (1)	6 (3)	
5% Dextrose	3 (75)	11 (73)	41 (26)	55 (31)	

* Not listed in MSD catalogue in 2009.

** Item should not be stocked at this facility level according to MSD catalogue in 2009 [37].

A number of items present at the facility were not functioning, most commonly refrigerators, telephones, blood pressure machines and stethoscopes (Table 1).

The availability of supplies and drugs was high (Table 1); however, several essential items for maternal care were found in fewer than half of facilities, such as suture kits (31%) and oxytocin (18%). There were instances of items being available in lower facilities that were not required at that level, such as manual vacuum aspirators.

Recorded stock outs of essential drugs and supplies for maternal and newborn care were common (Table 2). All but four of the sixteen items examined had been out of stock in the majority of facilities between July 1st and December 31st 2008. Items were most commonly out of stock in dispensaries. Thirteen facilities (9% of those stocking any antibiotic, one hospital and 12 dispensaries) had had a stock out of all parenteral antibiotics at least once in the last six months of 2008 (Table 2).

**Table 2:** Frequency of stockouts of supplies or drugs in facilities

	n/N* (%)facilities stocking item where item has recorded stock out between July 1^**st **^**and December 31**^**st **^**2008**			
**Supply item or drug**	**Hospitals**	**Health centres**	**Dispensaries**	**Total**
Examination gloves	2/4 (50)	6/15 (40)	80/154 (52)	88/173 (51)
Giving set – blood	1/4 (25)	1/2 (50)	15/19 (79)	17/25 (68)
Giving set – other fluids	1/4 (25)	6/15 (40)	56/103 (54)	63/122 (52)
Intravenous cannulae – size 16, 18 or 20	1/4 (25)	9/15 (60)	49/78 (63)	59/97 (61)
Blank partograph	1/4 (25)	7/14 (50)	60/125 (48)	68/143 (48)
Cord ligatures	2/4 (50)	8/15 (53)	71/138 (51)	81/157 (52)
Any antibiotic injection	1/4 (25)	0/14 (0)	12/127 (9)	13/145 (10)
Hydralazine injection	1/4 (25)	0/0 (0)	29/32 (91)	30/85 (35)
Magnesium Sulphate injection	1/4 (25)	4/11 (36)	58/100 (58)	63/115 (55)
Ergometrine injection	1/4 (25)	7/15 (47)	75/138 (54)	83/157 (53)
Oxytocin injection	1/4 (25)	6/9 (67)	36/52 (69)	43/65 (66)
Misoprostol tablets (100 or 200 μg)	1/2 (50)	1/6 (17)	17/23 (74)	19/31 (61)
Normal saline	1/4 (25)	4/12 (33)	49/79 (62)	54/95 (57)
Ringer’s lactate	1/4 (25)	6/13 (46)	55/87 (63)	62/104 (60)
10% Dextrose	1/3 (75)	3/5 (60)	25/30 (83)	29/38 (76)
5% Dextrose	1/4 (25)	3/12 (25)	42/66 (64)	46/82 (56)

*N Excludes facilities where drug was out of stock on day of survey.

### Staff experiences

#### Respondent characteristics

A total of 54 staff from 35 facilities participated in the FGDs. Participants were mainly female (11 male participants), median age 40 to 49 (range 24–60 years) and had been in their current position less than ten years (range 6 months to 30 years). Participant qualifications included clinical officers (N = 10), nurse midwives (N = 10), nursing officers (N = 8), public health nurses (N = 10) and maternal and child health aides (N = 3). IDIs were conducted with one member from three different CHMTs.

#### Staff experiences of providing care

FGDs and interviews were coded under three themes: Staff perceptions of stocks of drugs and supplies and equipment maintenance, coping mechanisms and effects of delayed repairs or running out of stock.

Staff perceptions of stocks of drugs and supplies and equipment maintenance Staff reported durations from three days to a year for supplies to be received or equipment repaired. Most commonly staff reported long durations, which increased the likelihood of stock outs:

At < facility name > we have a serious problem of not having ferrous sulphate. We have been out of stock for about a year now, and we informed the DMO office. They just tell us to wait because there is no stock. [FGD dispensary/health centre staff]

There were also reports of facilities receiving supplies which they did not request.

Facilities commonly received supplies and repaired items when supervisors visited, which meant that staff attributed responsibility to supervisors to provide this service:

They come for supervision; they should also bring all the things we requested. [FGD dispensary/health centre staff]

Supervisors felt that taking supplies when going for supervision was part of their role, as well as being efficient;

This is a good thing because we do this to minimize the costs of taking another car separately going to the same places. [IDI CHMT]

As well as problems with the suppliers, facilities reported receiving infrequent supervision visits.

CHMT staff reported that the low supervision frequency was due to transport challenges, many of which related to fuel supply and vehicle maintenance problems.

We have a problem here of maintaining regular visits due to a shortage of funds for fuel. We do receive fuel but it is not enough to cover all the movements. [IDI CHMT]

There were many complaints about MSD from both facility staff and supervisors:

You go to a facility and find there are no working tools, so you find it hard to assess them because they can’t do the duties to the required standard if they don’t have the working tools. And you keep on reporting with no action from the supplier. This really is a problem. [IDI CHMT]

Effects of delayed repairs or running out of stock

Inadequate equipment and supplies were reported to have caused delays in patients receiving services and increased work for staff, through the need to source items from other facilities, or through causing unnecessary referrals;

A lack of drugs is a problem, […] Many facilities come to the hospital to ask for drugs. [FGD Hospital staff]

There was a woman who wanted to deliver and I wanted to increase contractions but I didn’t have infusion, and the woman stayed at the facility for a long time, then I decided to refer the woman to < facility name>”. [FGD dispensary/health centre staff]

Staff reported mixed perceptions towards them from the community regarding supply or equipment problems. Some staff felt that patients seem to understand the problems that facilities face regarding supplies;

When we don’t have the equipment or drugs we apologise to them and they normally understand. [FGD dispensary/health centre staff]

More commonly staff felt that they were blamed by communities for the lack of drugs and supplies.

People in the community say that we want money, that’s why we don’t give out drugs or vaccines. [FGD dispensary/health centre staff]

They do not reach us in time and we get complaints from the community, as they think that perhaps we are selling the drugs. [FGD dispensary/health centre staff]

The issue of a lack of supplies or functioning equipment appeared to have a direct effect on staff morale and confidence to provide patient care.

We worry about how to tell the patients that we don’t have drugs or vaccines when they come here expecting that they will get them. [FGD dispensary/health centre staff]

A lack of equipment sometimes makes staff fear doing their job, because a lack of equipment when trying to save the lives of the patients makes staff lack confidence. [FGD dispensary/health centre staff]

Coping mechanisms

Facility staff reported many ways in which they adapted and improvised in order to continue providing patient care in the absence of functioning equipment or sufficient supplies.

I wanted to use a suction machine because the baby had asphyxia, but there was no suction machine and these days it is not allowed to do mouth to mouth resuscitation. I decided to take a syringe to draw the dirty stuff out of the baby’s mouth to help the baby. [FGD Hospital staff]

We don't have an examination bed at the facility, but we made a wooden table that we are using for examining pregnant women. [FGD dispensary/health centre staff]

For example the weighing scale for newborns is broken, and I am just using a sheet to hang the baby on the weighing scale for older children. [FGD dispensary/health centre staff]

Some staff reported that when trying to provide care in such situations, in some instances the alternative techniques were not recommended practice. In the following quote the alternative sterilisation method described shortens the life of equipment:

Today I was helping a woman to deliver and after delivering I had to sterilize equipment using ‘jik’ [bleach] and by boiling; instead of using ‘setamide’ which is recommended. [FGD dispensary/health centre staff]

We also found staff taking risks with their own health in order to provide maternity care:

Then the mother delivered the baby but the baby was not breathing well; and there was no resuscitation machine so I had to use mouth to mouth resuscitation to help the baby. However, it is a risk to my health as I can be infected easily. [FGD dispensary/health centre staff]

## Discussion

Drugs, supplies, and functioning equipment are all crucial for a facility to provide maternal care. Staff reported long delays in receiving supplies which meant they were unable to provide good maternity care. The reports of missing care items and frequent stock outs were corroborated by the facility survey.

The situation described by staff not only resulted in sub-optimal care for patients but also additional workload for staff through unnecessary referrals and the need to seek resources from neighbouring facilities. Resourceful practices by staff striving to continue to provide maternity care in this context had the potential to damage other functioning equipment or even their own health.

High turnover and low morale amongst staff of rural health facilities have been reported previously, and attributed to poor management and lack of recognition by supervisors [13,38]. Our study shows that poor maintenance of equipment and lack of supplies also impacts upon staff morale through pressures from supervisors and communities.

The survey findings reported are in line with other studies in the area. The continuing low availability of blood pressure machines in Lindi region highlighted in 2007 [25] was also found to be an issue in the six districts surveyed here (three of which were in Lindi Region). However a few items noted as declining in availability between 2002 and 2007, such as cord ligatures and diazepam, were quite widely available in our study.

### Strengths and limitations

The effects of insufficient drug stocks and missing or non-functioning equipment on facility and district-level staff have been well explored in this study. However, we did not explore the effects of logistic problems above the district level, or the main causes of poor drug and equipment availability. Factors arising in this study but not explored in detail included issues with the supply companies, reliance on relatively rare supervision visits to bring supplies, and causes of equipment malfunction. Frequently finding broken equipment during the survey suggests poor systems for routine maintenance and repair of equipment, which has been noted as a problem in similar studies [39], or the purchase of poor quality equipment. Although the Tanzania Service Provision Assessment Survey 2006 found that 88% of facilities in the Southern Zone had a system for the replacement or repair of small equipment [19], the experiences reported in this study question the functioning of such systems.

The survey focused on determining the presence or absence, and recent stock outs, of a number of key items for maternal care. However this list was not exhaustive and excluded non-medical items that are essential for the provision of care or maintenance of facilities, e.g. a functioning and fuelled car at the district level. We examined neither the amount of drugs in stock nor the use of stock levels (the quantity of remaining stock that prompts the facility to reorder). Limited supplies of drugs and poor use of stock levels have been found to be obstacles in providing care in other studies in Tanzania [24,40].

We used both qualitative and quantitative data collection methods. The qualitative data were collected from different levels of health facilities and staff qualifications using two qualitative methods in order to obtain a broad range of views and experiences. Neither qualitative data collector was linked to the health service in the region which allowed them to remain independent to facilitate open discussions. The FGDs were organized to allow those who were less senior or from small facilities to feel comfortable to express their views. However both data collectors were male, which, given the majority of participants were female, may have prevented some participants from speaking freely. A variety of cadres participated from many facilities to gain experiences from different care perspectives, but the participants and facilities were not selected randomly.

All facilities in the study area were visited, so the quantitative findings represent public sector facilities in the study area. However, many women do not deliver in health facilities [22,28]. First-line facilities are the main provider of ANC, but many deliveries take place in higher-level facilities and Mission facilities. Although nationally the proportion of deliveries in non-governmental facilities is low [22], two of the six hospitals in the study area are non-governmental, and other large non-governmental facilities border the study area. Together these facilities carry out a large number of deliveries, thus making a marked contribution to facility deliveries in the area. Therefore the external validity of our findings has limitations.

Findings from the quantitative data on the availability of drugs, supplies and functioning equipment, and frequency of stock outs matched the situation described by health workers in the FGDs and IDIs.

## Conclusions

The chronic situation of insufficient supplies and drugs, and broken or missing equipment for maternity care found in this survey affected patient care, and staff morale, health and workloads. Nevertheless, staff showed great resourcefulness to provide care in the challenging context.

Addressing supply and drug stock-outs and malfunctioning equipment is the responsibility of facility and district staff, but also beyond. The Tanzanian government is attempting to reduce supply chain problems by changing from supply distribution via CHMTs to direct delivery to facilities [41]. The new system needs to be monitored to determine if such changes will result in improvements in stocks and maintenance of equipment, without compromising regular supervision sessions that are essential for ensuring quality of care and motivated staff [18,42].

## Competing interests

The authors declare that they have no competing interests.

## Authors’ contributions

JAS, DS and MT were responsible for the study concept and design. Statistical analysis and interpretation was conducted by SP and CH. Qualitative analysis and interpretation was conducted by DS and TM. SP wrote the first draft, CH, JAS, FM, JJ revised the paper and contributed to discussion. All authors read and approved the final manuscript. SP acts as guarantor for the study.

## Pre-publication history

The pre-publication history for this paper can be accessed here:

http://www.biomedcentral.com/1472-6963/13/61/prepub
